# Association of polymorphisms in metastasis suppressor genes NME1 and KISS1 with breast cancer development and metastasis

**DOI:** 10.1186/s43046-020-00037-1

**Published:** 2020-05-27

**Authors:** Sarah Antar, Naglaa Mokhtar, Mahmoud Adel Abd elghaffar, Amal K. Seleem

**Affiliations:** 1 Medical Biochemistry Department, Faculty of Medicine, Mansoura University, Mansoura, 35516 Egypt; 2 Oncology Center, Mansoura University, Mansoura, Egypt

**Keywords:** Breast cancer, KISS1, Metastasis suppressor, NME1, Polymorphism

## Abstract

**Background:**

NME1 and KISS1 genes are two tumor metastasis suppressor genes, mapped to chromosomes 17q21.3 and 1q32 respectively. Here, we analyzed the association of EcoR1 (rs34214448—G/T) polymorphism in NME1 gene and 9 del T (rs5780218—A/-) polymorphism in KISS1 gene with breast cancer development and metastasis.

**Results:**

The study included 75 women newly diagnosed with breast cancer recruited from Oncology Center at Mansoura University Hospitals and 37 age-matched healthy female volunteers as a control group. DNA was extracted from peripheral blood samples and genotyping of rs34214448 and rs5780218 SNPs was carried out by PCR-RFLP technique. NME1 EcoR1 (rs34214448) polymorphism has a statistically significant association with breast cancer risk (*P* < 0.001). Most of breast cancer group (55%) had heterozygous (G/T) genotype while most of control group (95%) had homozygous wild (G/G) genotype (*P* < 0.0005). Also, KISS1 rs5780218 polymorphism has a statistically significant association with breast cancer risk. The wild (A/A) genotype was associated with lower risk of breast cancer (A/- + -/- vs. A/A: OR = 3.1, 95% CI = 1.15–8.36, *P* = 0.025). EcoR1 (rs34214448) polymorphism revealed a significant association with tumor stage and distant metastasis as patients. Carriers of the wild (G/G) genotype were more likely to present with advanced disease stages and distant metastasis.

**Conclusions:**

Both EcoR1 (rs34214448) polymorphism of NME1 gene and rs5780218 polymorphism of KISS1 gene revealed significant association with increased risk of breast cancer development. The (G/G) genotype of EcoR1 polymorphism was associated with higher risk of breast cancer metastasis.

## Background

Breast cancer is the most commonly diagnosed cancer in females worldwide, accounting for 25% of all female cancers. It is the leading cause of cancer-related deaths in less-developed countries, while it is the second cause of death after lung cancer in developed countries [1]. The incidence rate of breast cancer among women of the Middle-East and Northern Africa (MENA) region is 31.1% with mortality rate of 20.9% per 100,000 women [2]. In Egypt, breast cancer represents 32% of all female cancers with incidence rates of 48.8 per 100,000 women [3].

Metastasis represents the main cause of breast cancer-related mortalities. Only 5–10% of newly diagnosed breast cancer patients present with distant metastasis. However, about 30% of breast cancer patients diagnosed with early-stage disease are estimated to develop metastatic disease despite often months or even years later [4]. The 5-year survival rate of patients with localized disease is 99%, while it declines to 27% for those with more aggressive distant disease [5].

Metastasis-suppressor genes have the ability to inhibit metastasis without affecting the growth of the primary tumor [6]. They can inhibit different steps in the metastatic cascade. Those steps start when the primary tumor cells invade the complex physical barriers (including basement membrane, extracellular matrix, and the vasculature) of the primary site, intravasate and disseminate to distant organs via the lymphatic or vascular system. And finally, extravasation and colonization of the secondary site occur [7]. Several metastasis suppressor genes have been identified; NME/NM23 nucleoside diphosphate kinase 1 (NME1) and KiSS-1 metastasis-suppressor (KISS1) genes are two examples.

NME1 is a member of the nucleoside diphosphate (NDP) kinase family of proteins which is mapped to chromosome 17q21.3. It was the first identified tumor metastasis suppressor gene and affects the expression of genes involved in cell migration, apoptosis, and angiogenesis. The mechanisms underlying its metastasis suppressor activity are not fully understood. It exerts three different enzymatic activities that have potential anti-metastatic functions including nucleoside diphosphate kinase (NDPK) activity, histidine protein kinase activity (HPK), and a 5′–3′ exonuclease activity [8–10]. Reduced NME1 expression has been correlated with metastatic forms of different cancers including melanoma, breast, prostate, and colon cancers [11, 12]. Several studies have reported the association between NME1 gene polymorphisms and the risk of cancer development, prognosis, and survival rates [13].

The EcoR1 polymorphism (rs34214448) was first described in 1991 and occurs in intron 1 of NME1 gene. It is a bi-allelic polymorphism (G/T) with no associated amino acid change [14]. It has been analyzed in association with different cancer types including breast, gastric, lung, and gynecologic cancers. It revealed significant association with increased risk for non-small cell lung cancer [13], while it was linked to lower tendency to develop cervical cancer [15]. This could be explained by the differential expression of NME1 gene in different tissues and the variable regulatory mechanisms acting in different cancers [13]. A significant correlation between EcoR1 polymorphism and the risk of lymph node metastasis was found in breast and gastric cancers [16, 17].

KISS1 gene is a metastasis suppressor mapped to chromosome 1q32 [18]. It was identified in melanoma and breast cancer experimental models as a suppressor of metastasis that can inhibit chemotaxis and cell invasion [19, 20]. It encodes for kisspeptins which have a role in metastasis suppression [21]. Several studies reported the dual role for KISS1 gene acting as both promoter and suppressor of tumorigenesis and metastasis depending on the type of the tumor. This reveals the effect of different tumor environments on the action of this gene (e.g., steroidal milieu and the presence or absence of other signaling molecules that might facilitate suppressor or promoter pathways) [22]. KISS1 expression was found to be low in patients with distant metastasis of breast cancer and hepatocellular carcinoma [23, 24].

Along the KISS1 gene, at least 294 single nucleotide polymorphisms (SNPs) have been identified [25]. The 9 del T (rs5780218) polymorphism occurs at − 146 position of the 5′UTR of the mRNA transcript of KISS1 gene and it represents the deletion of an adenine (A) nucleotide. It has been correlated to the risk of breast cancer development among Mexican patients [26].

The aim of this study was to investigate the role of NME1 gene EcoR1 (rs34214448) and KISS1 gene 9 del T (rs5780218) polymorphisms in breast cancer. We analyzed their association with the risk of breast cancer development in a cohort of Egyptian population. In addition, we assessed their role in prediction of patients who are genetically predisposed to the development of metastatic disease.

## Methods

### Study participants

This case-control study was carried out in the Molecular Biology laboratory of the Medical Biochemistry Department, Faculty of Medicine, Mansoura University, Egypt. The study included 75 female patients newly diagnosed with breast cancer. They were recruited from the Oncology Center at Mansoura University Hospitals. Patients with other malignancy or cancer directed treatment (hormonal therapy, radiotherapy, or chemotherapy) were excluded from the study. The study also included a control group of 37 age matched, apparently healthy female volunteers, with no prior history of malignant disease.

Data of breast cancer patients were obtained from their medical records after they underwent the routine clinical and pathological investigations at the Oncology Center. Clinicopathological data included tumor stage, node metastasis, distant metastasis, and tumor markers status including estrogen receptor (ER), progesterone receptor (PR), human epidermal growth factor receptor 2 (Her2), and the cell proliferation marker (Ki-67). Clinical staging of breast cancer patients was carried out according to the American Joint Committee on Cancer (AJCC) staging system, eighth edition [27].

Ethical approval was obtained from the Institutional Review Board (IRB) of Faculty of Medicine, Mansoura University, Egypt (code number: MS/17.10.57). Written informed consent was obtained from all participants in the study.

### Sample collection

A sample of two milliliters (2 ml) of peripheral blood was obtained from all subjects by venipuncture, delivered to ethylenediaminetetraacetic acid (EDTA) tubes, properly labeled and stored at − 80 °C for further molecular analysis.

### DNA extraction

Genomic DNA was extracted from peripheral blood leucocytes using the GeneJET Whole Blood Genomic DNA Purification Mini Kit (Thermo Scientific, USA, Cat. no. #K0781).

### Genotyping of NME1 gene EcoR1 (rs34214448) and KISS1 gene 9 del T (rs5780218) polymorphisms

It was carried out by the technique of polymerase chain reaction-restriction fragment length polymorphism PCR (RFLP-PCR).

### Genotyping of NME1 gene EcoR1 (rs34214448) SNP

Genotypes of NME1 gene EcoR1 (rs34214448) polymorphism were determined using the forward primer 5′-CCCACCGTTTATTGGCTAG-3′ and the reverse primer 5′-CAACCCCCTTCATTTTACAA-3′. The PCR reaction was performed in a total volume of 25 μl, consisting of 12.5 μl of PCR master mix (2 ×), 2 μl of each primer (10 pmol/μl), DNA template (20 ng/μl), and nuclease-free water to get the final volume of 25 μl. The thermal cycler (Applied Biosystems, model 2720, Thermo Scientific, USA) was programmed according to the following amplification program: initial denaturation at 95 °C for 5 min, 35 cycles of denaturation at 95 °C for 30 s, annealing at 57 °C for 30 s, and extension at 72 °C for 30 s followed by final extension at 72 °C for 10 min.

The 151 bp PCR product was digested by EcoR1 restriction enzyme: (Enzynomics, Republic of Korea, Cat. no. R002S) at a temperature of 37 °C for 15 min with a total reaction volume of 30 μl. After digestion, 3% agarose gel stained with ethidium bromide was used to analyze the products. The enzyme cleaved T allele generating 2 fragments (82 bp and 69 bp), while the G allele was not digested.

### Genotyping of KISS1 gene 9 del T (rs5780218) SNP

Genotypes of KISS1 gene 9 del T (rs5780218) polymorphism were determined using the forward primer 5′-CCTTTGCCTGCCTGGATGCA-3′ and the reverse primer 5′-TGGGCCTGTGCTTGGAGACG-3′. The PCR reaction was performed in a total volume of 25 μl, consisting of 12.5 μl of PCR master mix (2 ×), 1 μl of each primer (10 pmol/μl), DNA template (20 ng/μl), and nuclease-free water to get the final volume of 25 μl. The thermal cycler (Applied Biosystems, model 2720, Thermo Scientific, USA) was programmed according to the following amplification program: initial denaturation at 95 °C for 5 min, 35 cycles of denaturation at 95 °C for 30 s, annealing at 65 °C for 30 s, and extension at 72 °C for 30 s followed by final extension at 72 °C for 10 min.

The 294 bp PCR product was digested by SMLI restriction enzyme: (New England BioLabs, USA, Cat. no. R0597S) at a temperature of 55 °C for 60 min with a total reaction volume of 30 μl. After digestion, 3% agarose gel stained with ethidium bromide was used to analyze the products. The enzyme cleaved A allele generating 2 fragments (224 bp and 70 bp), while the (-) allele was not digested.

### Statistical analysis

Hardy-Weinberg equilibrium (HWE) was assessed by applying an exact test to determine if the population was representative. Significant deviation from HWE was considered at the *P* < 0.05 level. For comparison of the allele and genotype frequencies between patients and controls, chi-square test (for cell values expected to be large) or Fisher exact tests (for cell values expected to be small, less than 5) were applied. The association between each SNP and breast cancer was evaluated under multiple inheritance models using the SNPStats software, a web-based program available at (https://www.snpstats.net/start.htm). We excluded 14 breast cancer patients with incomplete clinicopathological data from the analysis of the association between each SNP and tumor criteria. The SPSS software (version 25.0) was used for this analysis. A *P* value ≤ 0.05 was considered significant.

## Results

### Characteristics of the population studied

The clinicopathological and demographic characteristics of 75 breast cancer patients and 37 controls are presented in (Table 1). No statistically significant difference was found between the two study groups regarding age and menopausal status (*P* values were 0.862 and 0.256 respectively). In the breast cancer group, only 3 patients (4%) were diagnosed with stage I disease, 25 patients (33.3%) with stage II, 28 patients (37.3%) with stage III, and 19 patients (25.3%) with stage IV disease. Lymph node metastasis was detected in 52 patients (69.3%), while distant metastasis was detected in 19 patients (25.3%).

**Table 1:** Clinicopathologic and demographic characteristics of breast cancer patients and control groups

		Breast cancer patients (*n* = 75)	Control (*n* = 37)	*P* value
Age (years): median (IQR)		53 (41–64)	53 (38–63)	0.862
Menopausal status				
	Premenopausal	32 (42.7%)	20 (54.1%)	0.256
	Postmenopausal	43 (57.3%)	17 (45.9%)	
Tumor stage				
	Stage I	3 (4%)		
	Stage II	25 (33.3%)		
	Stage III	28 (37.3%)		
	Stage IV	19 (25.3%)		
Node metastasis				
	Yes	52 (69.3%)		
	No	23 (30.7%)		
Distant Metastasis				
	Yes	19 (25.3%)		
	No	56 (74.7%)		
ER status				
	Positive	56 (74.7%)		
	Negative	18 (24%)		
	Unknown	1 (1.3%)		
PR status				
	Positive	53 (70.7%)		
	Negative	21 (28%)		
	Unknown	1 (1.3%)		
HER2 status				
	Positive	29 (38.7%)		
	Negative	37 (49.3%)		
	Unknown	9 (12%)		
Ki-67				
	High	60 (80%)		
	Low	10 (13.3%)		
	Unknown	5 (6.7%)		

*IQR* interquartile range, *P* probability, *n* number, *ER* estrogen receptor, *PR* progesterone receptor, *HER2* human epidermal growth factor receptor 2, *Ki-67* cellular proliferation index

Data are presented as count and percent except where otherwise noted. Tests used: Mann-Whitney *U* test for data presented as median (IQR) and chi-square test for data presented as count and percent

The expected and observed frequencies of NME1 gene EcoR1 (rs34214448) SNP genotypes (G/G, G/T, T/T) were not statistically significant (*P* = 1). Also, the expected and observed frequencies of KISS1 gene 9 del T (rs5780218) SNP genotypes (A/A, A/-, -/-) were not statistically significant (*P* = 0.5). So, the study population was consistent with HWE for both SNPs.

### Correlation of NME1 gene EcoR1 (rs34214448) SNP with breast cancer

**Fig. 1:**
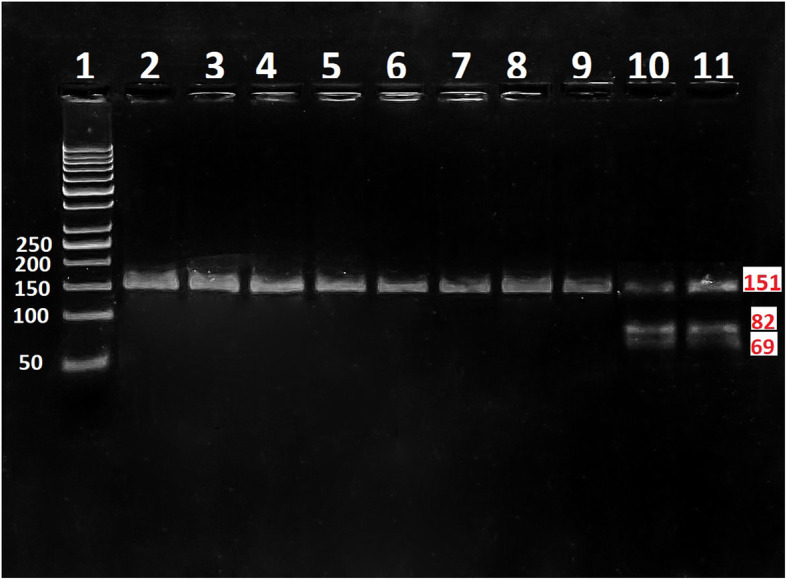
Three percent agarose gel electrophoresis of EcoR1 restriction enzyme products. Lane 1 contains a 50 bp DNA ladder; lanes 2–9 show the (G/G) genotype represented by one band of 151 bp; and lanes 10–11 show the (G/T) genotype represented by three bands of 152 bp, 82 bp, and 69 bp

The genotypic analysis of NME1 gene EcoR1 (rs34214448) polymorphism showed three genotypes as G/G, G/T, and T/T with different frequencies in (Fig. 1).

The genotype frequency in the healthy controls was 94.6%, 5.4%, and 0% for the G/G, G/T, and T/T genotypes respectively, while in the breast cancer group, it was 34.7%, 54.7%, and 10.7% for the G/G, G/T, and T/T genotypes respectively. The distribution of genotype frequency of NME1 EcoR1 (rs34214448) polymorphism showed a statistically significant difference between the two study groups (*P* < 0.0005). Most of the breast cancer group (54.7%) had G/T genotype, while most of the control group (94.6%) had G/G genotype.

The frequency of the major (G) allele was 97% and 62% for the control and breast cancer groups respectively, while the minor (T) allele frequency was 3% and 38% for the control and breast cancer groups respectively. There was statistically significant difference between the two study groups with regard to the distribution of allele frequency of NME1 EcoR1 (rs34214448) polymorphism (*P* < 0.0005) (Table 2).

**Table 2:** Genotype and allele frequencies of NME1 gene EcoR1 (rs34214448) and KISS1 gene 9 del T (rs5780218) SNPs in breast cancer patients and controls

	Breast cancer (*n* = 75)	Control (*n* = 37)	All (*n* = 112)	*P* value
NME1 gene EcoR1 (rs34214448)				
Genotype				
G/G	26 (34.7%) *	35 (94.6%) *	61 (54%)	< 0.0005 (P^MC^)
G/T	41 (54.7%) *	2 (5.4%) *	43 (38%)	
T/T	8 (10.7%) *	0 (0%) *	8 (7%)	
Allele				
G	93 (62%) *	72 (97%) *	165 (74%)	< 0.0005
T	57 (38%) *	2 (3%) *	59 (26%)	
KISS1 gene 9 del T (rs5780218)				
Genotype				
A/A	9 (12%)	11 (29.7%)	20 (18%)	0.068
A/-	55 (73.3%)	21 (56.8%)	76 (68%)	
-**/**-	11 (14.7%)	5 (13.5%)	16 (14%)	
Allele				
A	73 (49%)	43 (58%)	116 (52%)	0.183
-	77 (51%)	31 (42%)	108 (48%)	

Data are presented as count (percent). *P* value by chi-square test

(*)Indicates significant difference between the two groups

*n* number, *P* probability, *P*^*MC*^*P* (Monte Carlo)

NME1 EcoR1 (rs34214448) polymorphism was significantly correlated with breast cancer risk in all inheritance comparison models in the two study groups (all *P* values < 0.001). The log-additive model is the best inheritance model as it is the candidate that yields the strongest association with the smallest AIC and the minimum BIC (*P* < 0.0001, AIC = 103.4, BIC = 108.9). The minor T allele was associated with increased risk of breast cancer (OR = 28.02, 95% CI = 6.29–124.91, *P* < 0.0001) (Table 3).

**Table 3:** Logistic regression analysis of the association between NME1 gene EcoR1 (rs34214448) and KISS1 gene 9 del T (rs5780218) SNPs and breast cancer risk

SNP	Model	Genotype	Control (*n* = 37)	Breast cancer (*n* = 75)	OR (95% CI)	*P* value	AIC	BIC
NME1 gene EcoR1 (rs34214448)								
	Co-dominant	G/G G/T T/T	35 (94.6%) 2 (5.4%) 0 (0%)	26 (34.7%) 41 (54.7%) 8 (10.7%)	R 27.60 (6.11–124.59) NA (0.00–NA)	< 0.0001	105.4	113.6
	Dominant	G/G G/T-T/T	35 (94.6%) 2 (5.4%)	26 (34.7%) 49 (65.3%)	R 32.98 (7.35–148.08)	< 0.0001	104.1	109.5
	Recessive	G/G-G/T T/T	37 (100%) 0 (0%)	67 (89.3%) 8 (10.7%)	R NA (0.00–NA)	0.0096	139.4	144.8
	Over-dominant	G/G-T/T G/T	35 (94.6%) 2 (5.4%)	34 (45.3%) 41 (54.7%)	R 21.10 (4.73–94.16)	< 0.0001	115.8	121.3
	Log-additive	**–**	**–**	**–**	28.02 (6.29–124.91)	< 0.0001	103.4	108.9
KISS1 gene 9delT (rs5780218)								
	Co-dominant	A/A A/- -**/**-	11 (29.7%) 21 (56.8%) 5 (13.5%)	9 (12%) 55 (73.3%) 11 (14.7%)	R 3.2 (1.16-8.83) 2.69 (0.68–10.65)	0.077	143	151.2
	Dominant	A/A A**/**-,-**/**-	11 (29.7%) 26 (70.3%)	9 (12%) 66 (88%)	R 3.1 (1.15–8.36)	0.025	141.1	146.5
	Recessive	A/A- A**/**- -**/**-	32 (86.5%) 5 (13.5%)	64 (85.3%) 11 (14.7%)	R 1.1 (0.35–3.44)	0.87	146.1	151.5
	Over-dominant	A/A, -**/**- **A/**-	16 (43.2%) 21 (56.8%)	20 (26.7%) 55 (73.3%)	R 2.1 (0.92–4.79)	0.08	143.1	148.5
	Log-additive	**–**	**–**	**–**	1.83 (0.89–3.77)	0.095	143.3	148.8

*n* number, *R* reference category, *OR* odds ratio, *CI* confidence interval, *AIC* Akaike information criterion, *BIC* Bayesian information criterion, *NA* not applicable

Data are presented as count (percent). *P* value by standard (simple) logistic regression

Correlation of genotypes with tumor criteria was also carried out in the study population. The NME1 EcoR1 (rs34214448) polymorphism was significantly correlated with tumor stage and distant metastasis. Breast cancer patients with the wild (G/G) genotype were more likely to present with advanced tumor stages (III and IV) relative to patients with the mutant (T/T) genotype (39.5% vs. 2.6%, OR (95% CI) = 0.089 (0.009–0.873), *P* value = 0.038). The wild (G/G) genotype was also associated with higher risk of distant metastasis than the heterozygous genotype (G/T) (66.7% vs. 33.3%, OR (95% CI) = 0.25 (0.071–0.884), *P* value = 0.031). There was no significant correlation between the NME1 EcoR1 (rs34214448) polymorphism and other tumor criteria including lymph nodes’ involvement, ER, PR, HER2 receptors status, and KI-67 (all *P* values > 0.05) (Table 4).

**Table 4:** Association between genotypes of NME1 EcoR1 (rs34214448) polymorphism and tumor criteria in the breast cancer group

Genotype	Tumor criteria		OR (95%CI)	*P* value
	Tumor stage			
	Early (I and II) (*n* = 23)	Late (III and IV) (*n* = 38)		
G/G	8 (34.8%)	15 (39.5%)	R	
G/T	9 (39.1%)	22 (57.9%)	1.304 (0.410–4.145)	0.653
T/T	6 (26.1%)	1 (2.6%)	0.089 (0.009–0.873)	0.038
	LNs’ involvement			
	No (*n* = 19)	Yes (*n* = 42)		
G/G	8 (42.1%)	15 (35.7%)	R	
G/T	8 (42.1%)	23 (54.8%)	1.533 (0.473–4.971)	0.476
T/T	3 (15.8%)	4 (9.5%)	0.711 (0.127–3.993)	0.699
	Distant metastasis			
	**No** (n = 46)	**Yes** (n = 15)		
G/G	13 (28.3%)	10 (66.7%)	R	
G/T	26 (56.5%)	5 (33.3%)	0.25 (0.071–0.884)	0.031
T/T	7 (15.2%)	0 (0%)	0	0.999
	ER receptor status			
	Positive (*n* = 44)	Negative (*n* = 17)		
G/G	18 (40.9%)	5 (29.4%)	R	
G/T	21 (47.7%)	10 (58.8%)	1.714 (0.494–5.951)	0.396
T/T	5 (11.4%)	2 (11.8%)	1.44 (0.212–9.782)	0.709
	PR receptor status			
	Positive (*n* = 42)	Negative (*n* = 19)		
G/G	17 (40.5%)	6 (31.6%)	R	
G/T	20 (47.6%)	11 (57.9%)	1.558 (0.476–5.104)	0.464
T/T	5 (11.9%)	2 (10.5%)	1.133 (0.172–7.469)	0.896
	HER2 receptor status			
	Positive (*n* = 28)	Negative (*n* = 33)		
G/G	14 (50%)	9 (27.3%)	R	
G/T	11 (39.3%)	20 (60.6%)	2.828 (0.928–8.622)	0.068
T/T	3 (10.7%)	4 (12.1%)	2.074 (0.373–11.528)	0.405
	Ki-67 level			
	High (*n* = 54)	Low (*n* = 7)		
G/G	21 (38.9%)	2 (28.6%)	R	
G/T	26 (48.1%)	5 (71.4%)	2.019 (0.355–11.478)	0.428
T/T	7 (13%)	0 (0%)	0	0.999

*LN* lymph node, *ER* estrogen receptor, *PR* progesterone receptor, *HER2* human epidermal growth factor receptor 2, *Ki-67* cellular proliferation index, *R* reference category, *OR* odds ratio, *CI* confidence interval

Data are presented as count (percent). *P* value by standard (simple) logistic regression

### Correlation of KISS1 gene 9 del T (rs5780218) SNP with breast cancer

**Fig. 2:**
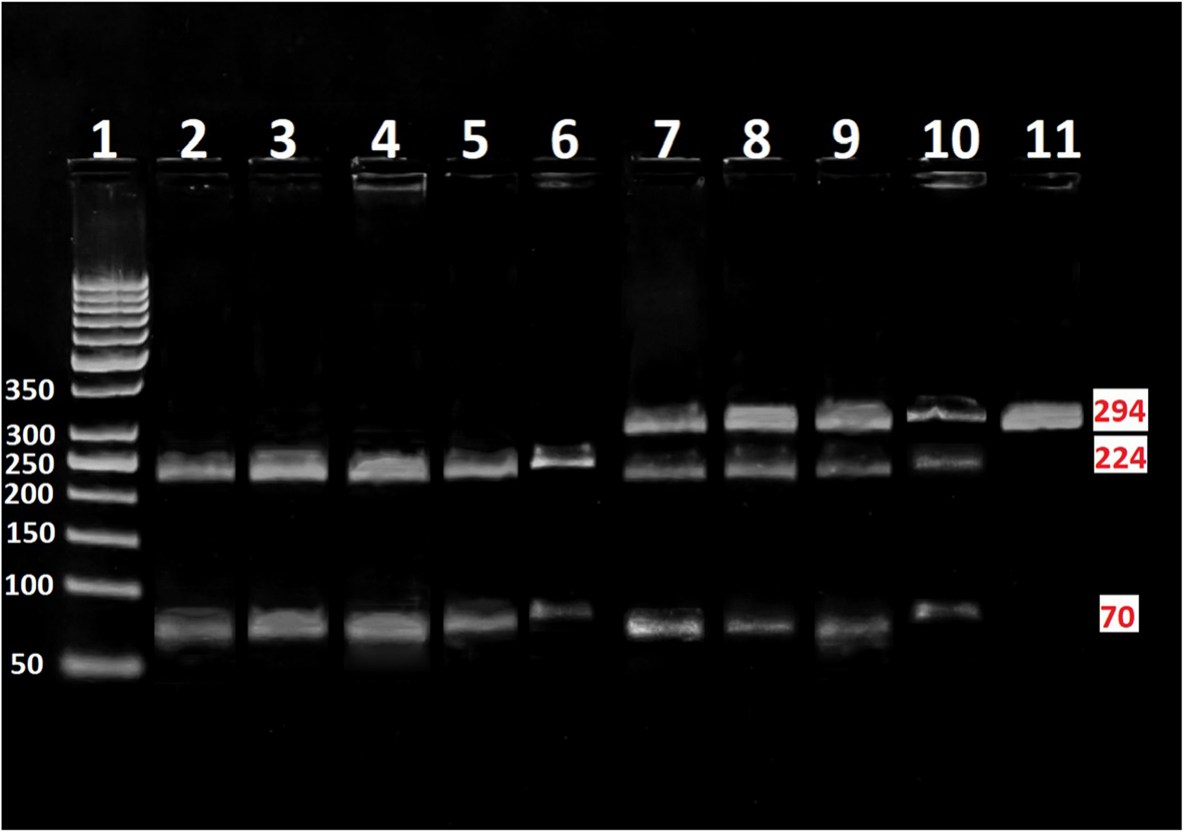
Three percent agarose gel electrophoresis of SmlI restriction enzyme products. Lane 1 contains a 50 bp DNA ladder; lanes 2–6 show the genotype (A/A) represented by two bands of 224 bp and 70 bp; lanes 7–10 show the (A/-) genotype represented by three bands of 294 bp, 224 bp, and 70 bp; and lane 11 shows the (-/-) genotype represented by one band of 294 bp

The genotypic analysis of KISS1 gene 9 del T (rs5780218) polymorphism showed three genotypes as A/A, A/-, and -/- with different frequencies in (Fig. 2).

The genotype frequency in the healthy controls was 29.7%, 56.8%, and 13.5% for the A/A, A/-, and -/- genotypes respectively; while for the breast cancer group, it was 12%, 73.3%, and 14.7% for the A/A, A/-, and -/- genotypes respectively. The A/- genotype was the most frequent genotype in breast cancer group (73.3%) and control group (56.8%).

The frequency of the major (A) allele was 58% and 49% for the control and breast cancer groups respectively, while the minor (-) allele frequency was 42% and 51% for the control and breast cancer groups respectively. The distribution of allele frequency of KISS1 rs5780218 polymorphism showed no statistically significant difference between the two study groups (*P* < 0.183) (Table 2).

KISS1 rs5780218 polymorphism was significantly correlated with breast cancer risk in the dominant inheritance model (A/- + -/- vs. A/A: OR = 3.1, 95% CI = 1.15–8.36, *P* = 0.025). The dominant model is the best inheritance model as it is the candidate that yields the strongest association with the smallest AIC and the minimum BIC (*P* = 0.025, AIC = 141.1, BIC = 146.5). The AA genotype was associated with lower risk for breast cancer under this model (Table 3).

Correlation of KISS1 rs5780218 genotypes with all tumor criteria in breast cancer group was also carried out in the study population and revealed no statistically significant association (all *P* values > 0.05) (Table 5).

**Table 5:** Association between genotypes of KISS1 rs5780218 polymorphism and tumor criteria in the breast cancer group

Genotype	Tumor criteria		OR (95%CI)	*P* value
	Tumor stage			
	Early (I and II) (*n* = 23)	Late (III and IV) (*n* = 38)		
A/A	2 (8.7%)	4 (10.5%)	R	
A/-	16 (69.6%)	30 (78.9%)	0.938 (0.155–5.686)	0.944
-**/**-	5 (21.7%)	4 (10.5%)	0.4 (0.047–3.424)	0.403
	LNs’ involvement			
	No (*n* = 19)	Yes (*n* = 42)		
A**/**A	3 (15.8%)	3 (7.1%)	R	
A**/**-	12 (63.2%)	34 (81%)	2.833 (0.502–15.987)	0.238
-**/**-	4 (21.1%)	5 (11.9%)	1.25 (0.158–9.917)	0.833
	Distant metastasis			
	No (*n* = 46)	Yes (*n* = 15)		
A**/**A	5 (10.9%)	1 (6.7%)	R	
A**/**-	34 (73.9%)	12 (80%)	1.765 (0.187–16.67)	0.62
-**/**-	7 (15.2%)	2 (13.3%)	1.429 (0.1–20.437)	0.793
	ER receptor status			
	Positive (*n* = 44)	Negative (*n* = 17)		
A/A	4 (9.1%)	2 (11.8%)	R	
A**/**-	32 (72.7%)	14 (82.4%)	0.875 (0.143–5.346)	0.885
-**/**-	8 (18.2%)	1 (5.9%)	0.25 (0.017–3.66)	0.311
	PR receptor status			
	Positive (*n* = 42)	Negative (*n* = 19)		
A**/**A	4 (9.5%)	2 (10.5%)	R	
A**/**-	30 (71.4%)	16 (84.2%)	1.067 (0.176–6.47)	0.944
-**/**-	8 (19%)	1 (5.3%)	0.25 (0.017–3.66)	0.311
	HER2 receptor status			
	Positive (*n* = 28)	Negative (*n* = 33)		
A/A	2 (7.1%)	4 (12.1%)	R	
A/-	21 (75%)	25 (75.8%)	0.595 (0.099–3.579)	0.571
-**/**-	5 (17.9%)	4 (12.1%)	0.4 (0.047–3.424)	0.403
	Ki-67 level			
	High (*n* = 54)	Low (*n* = 7)		
A/-	40 (74.1%)	6 (85.7%)	R	
A/A	6 (11.1%)	0 (0%)	0	0.999
-**/**-	8 (14.8%)	1 (14.3%)	0.833 (0.088-7.898)	0.874

*LN* lymph node, *ER* estrogen receptor, *PR* progesterone receptor, *HER2* human epidermal growth factor receptor 2, *Ki-67* cellular proliferation index, *R* reference category, *OR* odds ratio, *CI* confidence interval

Data are presented as count (percent). *P* value by standard (simple) logistic regression

## Discussion

Our study revealed that both NME1 gene EcoR1 (rs34214448) and KISS1 gene 9 del T (rs5780218) polymorphisms are linked to the risk of developing breast cancer.

Analysis of the allele and genotype distribution of the EcoR1 (rs34214448) polymorphism showed a significant difference between two studied groups. The major allele (G allele) predominates in the control group with a frequency of 97% vs. 62% for the breast cancer group, while the frequency of the minor allele (T allele) in the breast cancer group was higher than that in the control group (38% vs. 3%) (*P* < 0.0005). Most of the breast cancer patients had the heterozygous (G/T) genotype (55%), while most of the healthy control group had homozygous wild (G/G) genotype (95%) (*P* < 0.0005). The minor T allele was linked to increased risk of breast cancer development in the log-additive model of inheritance (OR = 28.02, 95% CI = 6.29-124.91, *P* < 0.0001).

In contrast to our results, Gutierrez Rubio et al. found no association between EcoRI polymorphism and the risk of breast cancer among Mexican patients. The heterozygous (G/T) genotype was the most common in both breast cancer and control groups (46.69% and 48.92% respectively) (*P* = 0.85). However, they suggested that analyzing its association with metastasis and evaluation of loss of heterozygosity in women with breast cancer may be of value [28]. Sample size difference and the use of tissue samples instead of blood sample may explain the discrepancies in the results.

One prior study on breast cancer patients in Kashmir, India, reported significant correlation between EcoR1 polymorphism and lymph node metastasis in patients with heterozygous genotype indicating an aggressive disease behavior [16]. EcoR1 polymorphism was also correlated to greater numbers of lymph node metastases in gastric cancer patients [17]. In addition, it showed an association with shorter recurrence-free survival in colorectal cancer patients with liver metastasis [29]. In contrast to the previous studies, EcoR1 polymorphism in our study cohort showed no association with lymph node metastasis. However, it was significantly correlated with tumor stage and distant metastasis. Breast cancer patients with the wild (G/G) genotype were more likely to present with advanced tumor stages (III and IV) relative to patients with the mutant (T/T) genotype (39.5% vs. 2.6%, OR (95% CI) = 0.089 (0.009–0.873), *P* value = 0.038). The wild (G/G) genotype was also associated with higher risk of distant metastasis than the heterozygous genotype (G/T) (66.7% vs. 33.3%, OR (95% CI) = 0.25 (0.071–0.884), *P* value = 0.031).

Our results display the contribution of EcoR1 (rs34214448) polymorphism to breast cancer development and metastasis. This is possibly due to its effect on the expression level of NME1 gene. Since, transcription factor-binding sites are located in the intron regions and promoter of NME1 genes, SNPs in those regions could change protein–DNA interactions and promoter activity affecting gene expression [17].

Analysis of the allele and genotype distribution of the KISS1 gene (rs5780218) polymorphism showed no significant difference between breast cancer and healthy control groups. The wild (A/A) genotype was associated with lower risk of breast cancer when compared to the (A/-) and (-/-) genotypes combined (A/- + -/- vs. A/A: OR = 3.1, 95% CI = 1.15–8.36, *P* = 0.025). Similarly, a prior study by Quevedo et al. reported a significant association between rs5780218 polymorphism and breast cancer risk among Mexican patients [26].

The KISS1 rs5780218 polymorphism had no significant correlation with any of the tumor criteria in the breast cancer group including lymph nodes’ involvement and distant metastasis.

The apparent discrepancies between the results of the current study and other reports on the genetic variations of NME1 and KISS1 genes may be related to sample size differences, ethnic, and population-specific variations which should be considered for interpretation of the results.

Our findings need to be validated via larger population-based studies. Further studies on haplotype blocks and well-designed functional experiments will be beneficial to elucidate the underlying mechanisms of those genetic variations in the development and progression of breast cancer. In addition, analysis of gene expression profiles of NME1 and KISS1 genes in blood and tissues may be helpful to develop a diagnostic and prognostic marker for breast cancer.

## Conclusions

In conclusion, this case-control study revealed a significant association between NME1 EcoR1 (rs34214448) polymorphism and the risk for breast cancer development. Carriers of the minor T allele had higher risk for breast cancer development, while the homozygous wild (G/G) genotype was associated with higher risk of metastasis. Also, the KISS1 rs5780218 polymorphism showed an association with the risk of breast cancer development with lower risk among carriers of the homozygous wild (A/A) genotype.

## Data Availability

All data generated or analyzed during this study are included in the manuscript.

## Abbreviations

NME1: Nucleoside diphosphate kinase 1
KISS1: KiSS-1 metastasis-suppressor
NDP: Nucleoside diphosphate
SNP: Single nucleotide polymorphism
ER: Estrogen receptor
PR: Progesterone receptor
Her2: Human epidermal growth factor receptor 2
Ki-67: Cell proliferation marker
AJCC: American Joint Committee on Cancer Staging System
EDTA: Ethylenediaminetetraacetic acid
HWE: Hardy-Weinberg equilibrium
OR: Odds ratio
